# Polygenic Risk Score for Metabolic Dysfunction-Associated Steatotic Liver Disease and Steatohepatitis: A Narrative Review

**DOI:** 10.3390/ijms26115164

**Published:** 2025-05-28

**Authors:** Tatsuo Kanda, Reina Sasaki-Tanaka, Hiroyuki Abe, Naruhiro Kimura, Tomoaki Yoshida, Kazunao Hayashi, Akira Sakamaki, Takeshi Yokoo, Hiroteru Kamimura, Atsunori Tsuchiya, Kenya Kamimura, Shuji Terai

**Affiliations:** 1 Division of Gastroenterology and Hepatology, Uonuma Institute of Community Medicine, Niigata University Medical and Dental Hospital, Uonuma Kikan Hospital, Minamiuonuma 949-7302, Japan; 2 Division of Gastroenterology and Hepatology, Graduate School of Medical and Dental Sciences, Niigata University, Niigata 951-8520, Japankhayashi@med.niigata-u.ac.jp (K.H.); saka-a@med.niigata-u.ac.jp (A.S.); hiroteruk@med.niigata-u.ac.jp (H.K.);; 3 Department of Gastroenterology and Hepatology, Faculty of Medicine, University of Yamanashi, Chuo 409-3898, Japan; a.tsuchiya@yamanashi.ac.jp; 4 Department of General Medicine, Niigata University School of Medicine, Niigata 951-9510, Japan; kenya-k@med.niigata-u.ac.jp

**Keywords:** MASLD, fat accumulation, liver fibrosis, MASH, cirrhosis, liver cancer, PRS, PNPLA3

## Abstract

Metabolic dysfunction-associated steatotic liver disease (MASLD) and metabolic dysfunction-associated steatohepatitis (MASH) are spreading worldwide as the most critical causes of cirrhosis and hepatocellular carcinoma (HCC). Thus, improving the screening and managing strategies for patients with MASLD or MASH is necessary. A traditional non-systemic review provided this narrative. Genetic variations associated with the development of MASLD and MASH, such as *PNPLA3*, *TM6SF2*, *GCKR*, *MBOAT7*, *MERTK*, and *HSD17B13*, were initially reviewed. *PNPLA3* genetic variants appeared to be strongly associated with the increased pathogenesis of MASLD, MASH, cirrhosis, and HCC. We also reviewed the useful polygenic risk score (PRS) for the development of MASLD, MASH, their related cirrhosis, and the occurrence of HCC. PRSs appeared to be better predictors of MASLD, MASH, the development of cirrhosis, and the occurrence of HCC in patients with MASLD or MASH than any single-nucleotide polymorphisms. RNA interference and antisense nucleotides against the genetic variations of *PNPLA3* and *HSD17B13* are also being developed. Multidisciplinary collaboration and cooperation involving hepatologists, geneticists, pharmacologists, and pathologists should resolve complicated problems in MASLD and MASH. This narrative review highlights the importance of the genetic susceptibility and PRS as predictive markers and personalized medicine for patients with MASLD or MASH in the future.

## 1. Introduction

The prevalence of metabolic dysfunction-associated steatotic liver disease (MASLD) among type 2 diabetes mellitus cases is high and growing. The majority of patients with MASLD and type 2 diabetes mellitus have metabolic dysfunction-associated steatohepatitis (MASH), including advanced fibrosis [1,2]. Improving the screening and management strategies for MASLD and MASH is required.

Recent guidelines and studies have defined and used MASLD and MASH instead of nonalcoholic fatty liver disease (NAFLD) and nonalcoholic steatohepatitis (NASH), respectively [3,4,5,6,7]. MASLD is diagnosed based on histological, imaging, or blood biomarker findings that suggest fat deposition in the liver, together with the existence of overweight/obesity, type 2 diabetes mellitus, or evidence of at least two metabolic risk abnormalities [4].

MASLD and MASH are important worldwide causes of liver fibrosis, cirrhosis, and hepatocellular carcinoma (HCC) because of substantial improvements in the prevention and treatment of viral hepatitis [8,9]. The annual cumulative incidence of HCC was reported to be ~2.6% in patients with MASH-cirrhosis [10]. Of note, MASLD/MASH may itself pose a risk factor for HCC, even in the absence of cirrhosis [11]. Ertle et al. reported that cirrhosis was detected in only 52.8% of patients with MASLD/MASH-associated HCC [11].

MASH is associated with an increased risk of hepatic complications, including cirrhosis, hepatic decompensation, and HCC, as well as extra-hepatic complications, including cardiovascular disease (CVD) and chronic kidney disease [12,13,14]. It has been reported that metabolic syndrome, overweight/obesity, central obesity, type 2 diabetes mellitus, dyslipidemia, insulin resistance, hypertension, a high-caloric/-fructose diet, minimal physical activity, sarcopenia, myosteatosis, gut dysbiosis, obstructive sleep apnea, and hypothyroidism are modifiable risk factors of MASLD [9].

Genome-wide association studies (GWASs) have revealed that single-nucleotide polymorphisms of genes play a supporting role in the diagnosis, treatment, and prognosis predictability in patients with liver diseases [15,16,17]. In general, common complex diseases are thought to be polygenic disorders, and several genetic variants are involved in the formation of these diseases [18,19]. The genetic risk is often assessed by the polygenic risk score (PRS), which is a weighted sum of the number of risk alleles an individual patient carries. The PRS is useful for risk predictions, diagnosis support, treatment decision making, and prognosis predictions [18,19].

An older age, the male gender, and genetic variations such as patatin-like phospholipase domain-containing protein 3 (*PNPLA3*), transmembrane 6 superfamily member 2 (*TM6SF2*), glucokinase regulator (*GCKR*), membrane-bound O-acyltransferase domain-containing 7 (*MBOAT7*), myeloid–epithelial–reproductive tyrosine kinase (*MERTK*), and hydroxysteroid 17-beta dehydrogenase 13 (*HSD17B13*) are considered non-modifiable risk factors of MASLD [9]. In this review, we describe the PRS for MASLD, MASH and their related HCC.

## 2. Representative Genetic Variants Involved in the Progression of MASLD/MASH

### 2.1. Patatin-like Phospholipase Domain-Containing Protein 3 (PNPLA3)

#### 2.1.1. Distribution and Intracellular Localization of PNPLA3

*PNPLA3* is expressed in various human tissues, but its highest level was found in the liver, including in hepatocytes and hepatic stellate cells (HSCs), followed by the retina, skin, adipose tissue, kidney, brain, and spleen [20,21,22].

*PNPLA3* is one of the independent single-nucleotide polymorphisms (SNPs) related to the liver enzyme levels. PNPLA3 is a transmembrane protein with phospholipase activity in the liver, and PNPLAs play biological roles in regulating adipocyte differentiation [23,24].

#### 2.1.2. Function of PNPLA3

PNPLA3 degrades polyunsaturated fatty acid lipid droplets in an adipose triglyceride lipase (ATGL)-independent manner [20,25]. *PNPLA3* encodes a triacylglycerol lipase and mediates triacylglycerol hydrolysis in adipocytes, and PNPLA3 has lipase activity towards triglycerides and retinol esters as well as acyltransferase activity on phospholipids [26].

*Carboxypeptidase N subunit 1* (*CPN1*) encodes arginine carboxypeptidase 1, which is a liver-expressed plasma metalloprotease [27]. *Endoplasmic reticulum lipid raft-associated 1* (*ERLIN1*) encodes a member of the prohibitin family of proteins and is defined as lipid-raft-like domains of the endoplasmic reticulum. *Component of inhibitor of nuclear factor kappa B kinase* (*NF-κß*) *complex (CHUK/IKK-α*) is a ubiquitously expressed serine threonine protein kinase that modulates the *NF-κß*-transcription-factor-dependent activation of several gene promoters, suggesting that this gene may be involved in insulin resistance [26,27]. Genetic variants of *SAMM50 sorting and assembly machinery component* (*SAMM50*) could lead to mitochondrial dysfunction, as SAMM50 is a subunit of the mitochondrial SAM translocase complex for the importation of proteins [27].

#### 2.1.3. PNPLA3 Genetic Variants and MASLD, MASH, Cirrhosis, and HCC

Serum liver enzyme levels are influenced by genetic and environmental factors [28]. Yuan et al. performed a GWAS and identified *CPN1 rs11597390-ERLIN1 rs11597086-CHUK rs11591741* on chromosome 10 and *PNPLA3 rs2281135-SAMM50 rs2143571* on chromosome 22 as two loci influencing the serum alanine aminotransferase (ALT) levels [27].

Romeo et al. reported that an allele in *PNPLA3* (rs738409: I148M) was strongly associated with increased hepatic fat levels (*p* = 5.9 × 10^−10^) and with hepatic inflammation (*p* = 3.7 × 10^−4^) by a GWAS, suggesting that this genetic variant may provide predictive information for the developmental risk of hepatic steatosis and liver injury [29].

*PNPLA3 genetic variants* have influence over the status of liver diseases, ranging from simple steatosis to MASLD, MASH, cirrhosis, and HCC [26,30]. The accumulation of PNPLA3 on hepatic lipid droplets is the basis of associated hepatic steatosis [31]. PNPLA3 (I148M) promotes steatosis [32].

Bril et al. reported that *PNPLA3* genetic variant carriers with MASLD are insulin resistant equal to non-carriers with MASLD at the level of liver, muscle, and adipose tissue [33]. In *PNPLA3* genetic variant carriers, early recognition, aggressive intervention, and improved insulin resistance should be required.

Interleukin 32 (IL32) mRNA expression was increased in steatotic liver disease and MASH samples solely in PNPLA3 I148M (rs738409 CG/GG) variant carriers, not in non-carriers (CC) [29]. The combination of a Helicobacter pylori infection and *G-allele PNPLA3* appeared to exacerbate MASLD severity compared to that of each individually [34]. PNPLA3 I148M results in hepatic lipid accumulation, the induction of lipotoxicity and lipo-apoptosis in hepatocytes, and the production of damage-associated molecular patterns (DAMPs), cytokines, and chemokines, all leading to the recruitment and activation of macrophages and HSCs, thereby promoting liver fibrosis [35].

The global frequency of PNPLA3 I148M varies, and this difference seems to parallel differences in the prevalence of MASLD in each region (Table 1) [36,37,38].

### 2.2. Therapies Targeting Patatin-like Phospholipase Domain-Containing Protein 3 (PNPLA3)

Nucleotide-based therapies targeting *PNPLA3 mRNA*, using antisense oligonucleotides (ASOs) or small interfering RNAs (siRNAs), are under development [38]. AZD2693, which is a liver-targeted ASO against *PNPLA3 mRNA*, potently reduced the PNPLA3 expression in human hepatocytes and the livers of mice [39].

In phase 1 trials, AZD2693 with a half-life of 14–33 days was well tolerated. AZD2693 successfully led to a knockdown of 89% *PNPLA3 mRNA* and reduced its proteins on hepatic lipid droplets. AZD2693 dose-dependently reduced the hepatic fat content and decreased the IL6 levels and C-reactive protein (CRP) [40]. A phase 2b study for histological assessments has been initiated, and it aims to evaluate the treatment effects of the hepatic silencing of *PNPLA3* on histological MASH and liver fibrosis in patients with the PNPLA3 148M risk allele variant [41].

An in vitro study demonstrated that the efficacy of resmetirom, a thyroid hormone receptor beta agonist that was recently approved by the FDA for the treatment of MASLD, was influenced by *PNPLA3 variants* [42]. Further studies will be needed.

### 2.3. Transmembrane 6 Superfamily Member 2 (TM6SF2)

TM6SF2 is involved in the regulation of lipid metabolism. TM6SF2 normally acts to promote very-low-density lipoprotein (VLDL) secretion in mice, and the increased hepatic triglyceride content (HTGC) associated with the TM6SF2-167Lys variant leads to a reduction of TM6SF2 function in humans [43]. An increase in HTGC together with a decrease in plasma cholesterol and triglycerides is consistent with a defect in VLDL secretion, suggesting that TM6SF2 regulates the hepatic VLDL secretion pathway. TM6SF2 is expressed predominantly in the human liver and intestine, and it is located in the endoplasmic reticulum and the endoplasmic reticulum–Golgi intermediate compartment of human liver cells [44].

The Dallas Heart Study (DHS), an exome-wide association study on the hepatic triglyceride content, showed that two sequence variants in *PNPLA3* (rs738409 and rs2281135) had the lowest *p*-values (4.0 × 10^−16^ and 6.9 × 10^−12^, respectively), as did a variant (rs58542926) in TM6SF2 (*p* = 5.7 × 10^−8^) [43]. The Glu167LysTM6SF2 variant was also related to higher ALT levels and lower levels of low-density lipoprotein (LDL) cholesterol, triglycerides, and alkaline phosphatase [43].

Huang et al. reported that, using the Penn Medicine Biobank (PMBB) whole-exome sequence (WES) data (n > 40,000) and UK Biobank (UKB) WES data (n > 200,000), *missense variants in TM6SF2* (E167K, L156P, P216L) were associated with an increased risk of clinically diagnosed and imaging-proven hepatic steatosis, independent of the *PNPLA3* I48M risk allele and hepatitis B/C (*p* < 0.001), and that *TM6SF2* E167K homozygotes had a significantly increased risk of steatotic liver disease (odds ratio [OR]: 5.38, *p* < 0.001), steatohepatitis (OR: 5.76, *p* < 0.05), and HCC (OR: 11.22, *p* < 0.0001). In addition, carriers of *TM6SF2* E167K are at a 3-fold increased risk of MASH (OR: 2.75, *p* < 0.001) [45].

Seko et al. studied 1304 Japanese patients with biopsy-proven MASLD. They showed that, during the follow-up period of 8.1 years, there were 96 liver-related events and 53 deaths [46]. They found that the *TM6SF2* and *GCKR genotypes* were associated with the development of liver-related events [46]. Zhang et al. fed hepatocyte-specific Tm6sf2 knockout mice with a high-fat/high-cholesterol (HFHC) diet or a diethylnitrosamine-plus-HFHC diet to induce MASLD-HCC, and they concluded that hepatic TM6SF2 protects against MASLD-HCC and activates cytotoxic CD8+ T cells via the *NF-κß*-IL6 axis [47]. TM6SF2 may play a protective role against the occurrence of HCC in MASLD patients.

### 2.4. Glucokinase Regulator (GCKR)/Glucokinase Regulatory Protein (GKRP)

Glucokinase (GK) plays a central role in glucose metabolism. The glucokinase regulator (GCKR)/GK regulatory protein (GKRP) regulates GK with nuclear sequestration at low plasma glucose levels. GK enhances hepatic glucose uptake and glycogen synthesis [48]. The excessive activity of hepatic GK may enhance the capacity of glycogen synthesis with the extreme formation of triglycerides. An impaired GK-GKRP interaction may worsen lipid profiles and increase the risks of MASLD and coronary artery disease [48].

P446L-GCKR reduces regulation by physiological concentrations of fructose-6 phosphate, leading indirectly to increased GCK activity [49]. Maffeis et al. reported that the prevalence of MASLD was 27.5% (67/244) in children and adolescents with type 1 diabetes mellitus [50]. Thus, MASLD is a common condition in children and adolescents with type 1 diabetes mellitus.

The *GCKR* rs1260326 *gene variant* is related to greater glycolysis, which increases hepatic de novo lipogenesis [51]. The *GCKR* rs1260326 *gene variant* is also related to hepatic fat accumulation along with high VLDL and triglyceride levels [52]. GCKR and PNPLA3 act together to convey susceptibility to fatty liver in obese young adults. Among 4804 adult non-Hispanic white (NHW), non-Hispanic black (NHB), and Mexican American (MA) participants (1825 NHW, 1442 NHB, and 1537 MA; 51.7% women; mean age at examination, 42.5 y), the G allele of *PNPLA3* rs738409 and the T allele of *GCKR* rs780094 were associated with hepatic steatosis with a high level of ALT (odds ratio [OR]: 1.36, *p* = 0.01, and OR: 1.30, *p* = 0.03, respectively) [53].

### 2.5. Membrane-Bound O-Acyltransferase Domain-Containing 7 (MBOAT7)

MBOAT7 is a membrane integral protein with acyltransferase activity. Several GWASs have demonstrated that *MBOAT7* rs641738 is associated with a risk of MASLD in individuals of European descent, as well as alcoholic-associated cirrhosis and liver fibrosis in chronic hepatitis C patients [54,55,56]. MPOAT7 was found to be highly expressed in the liver. *MBOAT7* rs641738 was associated with an increased hepatic fat content in 2736 participants from the DHS cohort, who also underwent proton magnetic resonance spectroscopy to measure the HTGC, and in 1149 European patients from the liver biopsy cross-sectional cohort to diagnose liver disease and disease severity [54]. There was an association between the MBOAT7 rs641738 variant and the development and severity of MASLD in individuals of European descent [54].

Bush et al. also observed variants in the *MBOAT7* (*p* = 1.03 × 10^−9^) and *TM6SF2* (*p* = 7.89 × 10^−10^) genes as well as rs738409 in *PNPLA3* (*p* = 1.54 × 10^−48^) as an important risk locus for alcohol-related cirrhosis [55]. *MBOAT7* rs641738 polymorphism is a risk variant for inflammation and fibrosis of the liver in chronic hepatitis C patients [56].

Alharthi et al. reported that MBOAT7 is a negative regulator of toll-like receptor (TLR) signaling. A disturbance in TLR signaling leads to tissue damage and a deficiency of MBOAT7 in macrophages, as observed in MASLD; this alters the membrane phospholipid composition in association with a redistribution of arachidonic acid toward proinflammatory eicosanoids, the induction of endoplasmic reticulum stress, and mitochondrial dysfunction [57]. The common *MBOAT7 variant* rs641738 C > T is a risk factor for MASLD and MASH, and this variant leads to the decreased expression of the phospholipid-remodeling enzyme MBOAT7 (LPIAT1) [58]. Changes in hepatocyte phospholipids due to MBOAT7 loss-of-function result in the increase in a cholesterol trafficking pathway that upregulates TAZ (WWTR1) and the TAZ-induced profibrotic factor Indian hedgehog (IHH) [58]. *MBOAT7* rs641738 gene polymorphism is associated with both the severity of liver fibrosis and inflammation [59].

### 2.6. Myeloid-Epithelial-Reproductive Tyrosine Kinase (MERTK)

MERTK is a transmembrane protein that has two fibronectin type III domains—two immunoglobulin-like domains and one tyrosine kinase domain. Clinically significant fibrosis (stage F2—F4) was observed in 19% of patients with *MERTK* rs4374383 AA compared to 30% of those with *MERTK* rs4374383 GG/GA (OR: 0.43, CI: 0.21–0.88, *p* = 0.02) [60]. The *MERTK* rs4374383 AA genotype is associated with the lower intrahepatic expression of MERTK and is protective against F2-F4 liver fibrosis progression in MASLD patients. The inhibition of MERTK activity induces the apoptosis of HSCs and decreases procollagen expression [60].

In NASH mice, MERTK cleavage by ADAM metallopeptidase domain 17 (ADAM17) in liver macrophages decreases during the MASLD-to-MASH transition, and mice with a cleavage-resistant MERTK mutant have increased MASH fibrosis [61]. Similar to the findings with bone marrow-derived macrophages, all-trans retinoic acid (ATRA) suppressed p-AKT and increased p-P38, ADAM17 activity, and MERTK cleavage in isolated Kupffer cells [61,62]. The TAM (TYRO3, AXL, and MERTK) regulation of liver fibrogenesis and the inflammation mechanisms underlying MASH, cirrhosis, and HCC has recently been revealed [63]. Protein S (PROS) and growth-arrest-specific 6 (GAS6) interact with TAM. GAS6-AXL signaling plays a role in liver regeneration. The pharmacological inhibition of AXL seems to be more efficient at reducing liver fibrosis progression [63]. The AXL kinase targeting of liver immune cells could diminish liver inflammation and fibrosis in experimental MASH [64].

### 2.7. Hydroxysteroid 17-Beta Dehydrogenase 13 (HSD17B13)

HSD17B13 plays a role in the positive regulation of the lipid biosynthetic process. A splice variant of HSD17B13 (rs72613567: TA), which encodes the hepatic lipid droplet protein hydroxysteroid 17-beta dehydrogenase 13, was associated with a reduction in ALT and aspartate aminotransferase (AST) levels (*p* = 4.2 × 10^−12^ and *p* = 6.2 × 10^−10^, respectively). A loss-of-function variant in HSD17B13 was related to a reduced risk of progression from steatosis to steatohepatitis [65].

The *HSD17B13* rs72613567: TA variant induces a splice donor site mutation, resulting in the production of a truncated, non-functional HSD17B13 decoding protein [66]. The knockdown of HSD17B13 in high-fat-diet obese mice could lead to the alleviation of MASLD through the regulation of fatty acid and phospholipid metabolism [67]. HSD17B13 seems to be one of the therapeutic targets for MASLD and the development of liver fibrosis. These genetic variants, which are associated with lipid droplets in the liver, hepatic VLDL secretion, and de novo *lipogenesis*, seem to be involved in the pathogenesis of MASLD and MASH. The effects of these representative genes on steatotic liver diseases are shown in Table 2.

### 2.8. Other Genes

*Ectonucleotide pyrophosphatase/phosphodiesterase 1* (*ENPP1*) (rs1044498, *p* = 0.0091), *PNPLA3* (rs738409, *p* = 2.80 × 10^−6^), and *GCKR* (rs780094, *p* = 0.0281) were significantly associated with pediatric MASLD [68]. ENPP1 has been found to be associated with insulin resistance and lipid accumulation in the liver [69].

MASLD patients without fibrosis had a higher frequency of *interferon λ3* (*IL28B*) rs12979860 TT and rs12980275 GG genotypes compared with MASLD patients with fibrosis (*p* < 0.005) [70]. The IL28B genotype is associated with a response to interferon alpha and ribavirin therapy in patients with chronic hepatitis C [15,16,17]. *Lysophospholipase-like 1* (*LYPLAL1*) rs12137855 was associated with MASLD in a Chinese Han population [71]. LYPLA1 encodes lysophospholipase-like protein 1, which is associated with hepatic lipid and glucose metabolism [72,73].

Chouik et al. reported that the combination of a heterozygous *apolipoprotein* B (*APOB*) gene mutation with the *PNPLA3* and *TM6SF2* variants accelerated steatotic liver disease, cirrhosis, and the occurrence of HCC [74]. The hepatic secretion of VLDL is restrained by the inhibition of microsomal triglyceride transfer protein (MTP; gene name, *MTTP*), leading to an increase in hepatic steatosis and a reduction in serum lipids and apoB [75]. MTTP I128T is associated with a reduction in hepatic steatosis, plasma lipids, and apoB. Rare inactivating variants in the *APOB* or *MTTP* genes were observed in 0.8% of individuals with steatosis and conferred a more than 6-fold risk (*p* < 2 × 10^−5^) [76].

*Lipin 1* (*LPIN1*) rs13412852 polymorphism is related to the severity of liver damage and liver fibrosis progression in children with histological MASLD [77]. Lipin 1 is essential in lipid metabolism. Uncoupling protein 2 (*UCP2*) rs695366-G (HR for steatotic liver disease: A/G 0.63, *p* = 0.002, G/G HR: 0.50, *p* = 0.04) appeared as the most critical genetic risk factor for steatotic liver diseases post-liver transplantation [78]. Lipids increase reactive oxygen species (ROS) and induce UCP2 in hepatocytes [79]. These other genes may be involved in the development of MASLD, MASH, and HCC.

## 3. Polygenic Risk Score (PRS) for the Progression of MASLD/MASH

### 3.1. PRS for ALT Elevation, Hepatic Fat Accumulation, and Liver Fibrosis

PRSs consist of measures of genetic susceptibility with clinical utility in human disease and health. They augment risk predictions of prognoses, including the development of cirrhosis and the occurrence of HCC; refine diagnoses; guide treatment choices; make clinical trials more efficient; and improve public health [80]. The PRS is a road for an individual’s genetic liability to a disease. It is calculated based on their genotype profile and relevant GWAS data [81]. Further work with PRS-based risk assessments and development is needed in the clinical practice of MASLD, a complex disease that seems to be contributed to by larger sets of genetic variants [82,83].

The PRSs associated with MASLD and MASH are shown in Table 3. Larrieta-Carrasco et al. examined the association between 288 SNPs identified in GWASs and the risk of elevated transaminase levels in an admixed Mexican-Mestizo sample of 178 cases of MASLD and 454 healthy controls [84]. The rs2896019, rs12483959, and rs3810622 SNPs in PNPLA3 and rs1227756 in *collagen type XIII alpha 1 chain* (*COL13A1*) were associated with elevated ALT levels (≥40 IU/L). A PRS based on six SNPs in the *adiponectin*, *C1Q and collagen domain-containing* (*ADIPOQ*), *COL13A1*, *PNPLA3*, and *SAMM50* genes was also associated with the elevation of the ALT level [84]. 

Stender et al. examined 4018 British children and adolescents from the Avon Longitudinal Study of Parents and Children (ALSPAC) cohort [85]. Genetic risk factors for MASLD were associated with higher ALT levels, beginning in childhood and throughout adolescence and early adulthood. The ALT levels increased with age and were highest among young adults. The *p*-value for the interaction between time and genome-wide ALT-PRS, including *PNPLA3* rs738409, *TM6SF2* rs58542926, and *HSD17B13* rs72613567, on inverse normalized ALT was 1.5 × 10^−4^ [85]. *PNPLA3* and *other gene variant*-based PRSs for the elevation of ALT levels have been reported in both children and adults (Table 3) [84,85].

Dongiovanni et al. reported *PNPLA3* and *other gene variant*-based PRSs for hepatic fat accumulation and liver fibrosis (Table 3) [86]. They concluded that interventions aimed at reducing hepatic steatosis seem to have long-term beneficial effects on liver disease as well as potentially on insulin resistance in patients with MASLD. PRS-5 based on variations in *PNPLA3*, *TM6SF2*, *GCKR*, *MBOAT7*, and *HSD17B13* is also useful for the prediction of liver fibrosis (Table 3) [87].

### 3.2. PRS for MASLD and MASH

There have been several PRSs for MASLD [88,89,90,91,92] and MASH [93,94,95] (Table 3). Miao et al. accurately estimated the MASLD status in the UKB, identified 90 GWAS MASLD loci, built the MASLD PRS, and discovered a significant causal effect of MASLD on coronary artery disease [88]. Ge et al. reported that, during a median follow-up of 12.4 years, 3201 incidental MASLD cases were confirmed from the UKB (N = 338,087) [89]. The PRS with variants in *PNPLA3*, *TM6SF2*, *MBOAT7*, and *GCKR* predicts MASLD with the HR at 1.78 (95% CI, 1.60–1.98) (Table 3).

Fu et al. reported that positive associations of the PRS for type 2 diabetes mellitus with gastritis, duodenitis, and MASLD were also observed (Table 3) [90]. The 20-year cumulative incidence of liver-related outcomes in MASLD was investigated, with the results showing a mean follow-up of 12.1 years (1,128,069 person-years) and a crude incidence rate of liver-related outcomes in MASLD of 0.97/1000 person-years, resulting in an intermediate genetic risk (OR, 1.13 (95% CI, 1.02–1.25)) and a high genetic risk (OR, 1.42 (95% CI, 1.26–1.59)) (Table 3) [91]. Giardoglou et al. also demonstrated strong evidence that the PRS is a powerful prediction model for MASLD, using UKB data to assess an individual’s genetic liability to MASLD [92].

Kim et al. examined the PRS in relation to dyslipidemia among 48,263 South Koreans (17,064 men and 31,199 women) and validated simple indexes for MASLD and MASH as predictors of dyslipidemia using the PRS in East Asian men [93]. Gao et al. showed that the nomogram, which includes sex, metabolic syndrome, a homeostatic model assessment for insulin resistance (HOMA-IR), AST levels, and the *PNPLA3* and *HSD17B13 genotypes*, is useful, and that individualized PRSs can identify MASH in the Eastern Asia region [94].

Bridi et al. prospectively examined the PRS of the sum of risk alleles in PNPLA3, TM6SF2, and SERPINA1 minus the protective variant in HSD17B13 among 382 patients aged ≥ 50 years and with type 2 diabetes mellitus, and showed that a higher PRS is associated with an increased risk of cirrhosis (*p* = 0.037) and also that a high PRS is associated with an increased risk of advanced cirrhosis among those with a fibrosis-4 index <1.3 (*p* = 0.036) [95]. They recommended the addition of an assessment of genetic risk to screen at-risk populations, as this may improve risk predictions (Table 3). Thus, the PRS for MASLD and MASH is useful, and further studies are now under way.

### 3.3. PRS for Severe Liver Diseases, Such as Cirrhosis and HCC in MASLD MASH

In general, it is difficult to predict severe liver diseases, including the development of cirrhosis and the occurrence of HCC in patients with MASLD or MASH. So, PRSs are also useful for the prediction of these conditions in patients with MASLD or MASH (Table 3) [46,95,96,97,98,99,100,101]. De Vincentis et al. examined the UKB cohort (n = 266,687) and showed that the HFC-PRS (including *PNPLA3*, *TM6SF2*, *MBOAT7*, and *GCKR*) was highly related to the risk of severe liver disease in the overall population (age–sex-adjusted HR (aHR) for a 1 SD increase: 1.25; 95% CI, 1.16–1.35; *p* = 8.9 × 10^−9^) [96].

Gellert-Kristensen et al. showed that the PRS for fatty liver disease confers up to a 12-fold higher risk of cirrhosis and up to a 29-fold higher risk of HCC in individuals from the general population [97]. The PRS may accurately help to detect HCC and stratify the HCC risk in individuals with dysmetabolism [98]. The PRS could predict the risk of HCC in patients with MASLD/MASH in both Eastern and Western countries (Table 3) [99,100,101].

Thomas et al. used resources from 24,333 participants of the Singapore Chinese Health Study (SCHS) and examined the association between the PRS for hepatic fat (HFC-PRS) and the HCC risk [99]. The HFC-PRS was strongly associated with a statistically significantly higher risk of HCC in the East Asian population (p trend  <  0.001) (Table 3). A total of 1644 patients with cirrhosis from two prospective cohort studies with genotyping, the Texas Hepatocellular Carcinoma Consortium cohort (THCCC) and the Houston Veterans Administration cirrhosis surveillance cohort (HVASC), were examined [100]. The PRS (high-risk variants in *PNPLA3-MBOAT7-TM6SF2-GCKR*) exhibited by cirrhosis patients in the highest tertile corresponded to a 2-fold higher risk of HCC (HR = 2.05; 95% CI, 1.22–3.44) (Table 3). The PRS may enhance the risk prediction for HCC in U.S. cirrhosis patients [100].

Xiao et al. found 16 SNPs (*GCKR*, 2:27748992:AT:A; rs6731688; *LOC124905962*, rs62106258; *MON1A*, 3:49959570:CA:C; *MLXIPL*, rs17145750; *LPL*, rs2119690; *BDNF/BDNF-AS*, rs11030108; *ZPR1*, rs964184; *FAIM2*, rs7132908; *EXOC3L4*, rs2274685; *FTO*, rs11075985; *APOBR*, rs40831; *RP11-795H16.2*, 18:57850927:GTCT:G; rs538303513; BCRP3, rs116946885; GGT1, rs3859862) at genome-wide significance levels for MASLD, which was duplicated in the replication cohort [101]. Differences were found after comparing these SNPs with the results of NAFLD-related genetic variants. A high PRS amplified the impact of MASLD on severe liver disease and the extrahepatic outcomes [101] (Table 3).

Of interest, the PRSs for the microbiota [102], body mass index (BMI) [103], and brain function [104] in patients with MASLD or MASH have been reported. Pirola et al. confirmed the evidence that genetic variation may influence the liver microbial DNA composition (Table 3) [102]. Data from 2185 participants in a Greek population were used, with the results showing a 343-single-nucleotide polymorphism PRS yielding an R^2^ = 0.3241 (beta = 1.011, *p* = 4 × 10^−193^) for BMI [103]. Guo et al. observed that liver diseases were risk factors for brain disorders, with genetic and biochemical associations contributing to these risks, using a prospective cohort with data from 492,059 participants in the UKB (Table 3) [104].

## 4. Discussion

In this review, genetic variants, which are involved in the progression of MASLD/MASH and PRSs, were considered useful for the clinical practice of MASLD and MASH, although other biomarkers may replace these genetic markers in the future. The PRS seems to be superior to single-gene polymorphisms. Genetic variations (*PNPLA3*, *TM6SF2*, *GCKR*, *MBOAT7*, *MERTK*, and *HSD17B13*) are one of the non-modifiable risk factors for metabolic dysfunction-associated fatty liver disease (MAFLD) and MASLD [9,14,105,106,107]. Clinicians at large volume centers may consider assessing the genetic risk profile, such as PNPLA3 I148M and/or PRS, to personalize risk stratification. Genetic variants in *PNPLA3* and *HSD17B13* may be one of the targets to be knocked down by RNA interference and antisense oligonucleotide therapies [39,40,108], leading to the prevention of the development to severe liver diseases in patients with MASLD/MASH. Further investigations are needed.

**Figure 1:**
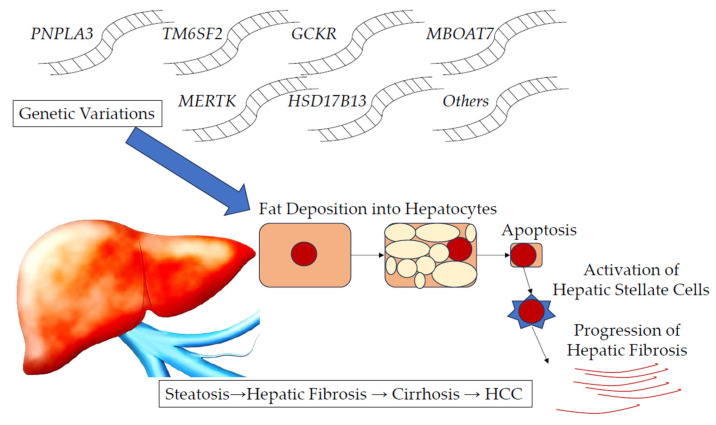
Effects of genetic variations in *PNPLA3*, *TM6SF2*, *GCKR*, *MBOAT7*, *MERTK*, *HSD17B13*, etc., on the liver. Fat deposition into hepatocytes, apoptotic hepatocyte death, and the activation of hepatic stellate cells occur, causing hepatic steatosis, fibrosis, cirrhosis, and hepatocellular carcinoma (HCC) [8,110]. *PNPLA3*, *patatin-like phospholipase domain-containing protein 3*; *TM6SF2*, *transmembrane 6 superfamily member 2*; *GCKR*, *glucokinase regulator*; *MBOAT7*, *membrane-bound O-acyltransferase domain-containing 7*; *MERTK*, *myeloid–epithelial–reproductive tyrosine kinase*; *HSD17B13*, *hydroxysteroid 17-beta dehydrogenase 13*.

Genetic variations accelerate abnormalities in glucose and lipid metabolism as well as in microbiota [8,109]. These accelerate hepatic fat deposition/accumulation, resulting in the apoptosis of hepatocytes [110]. Apoptotic hepatocyte death stimulates immune cells and HSCs toward the progression of fibrosis in the liver through the production of inflammasomes and cytokines. ROS, oxidative stress, and endoplasmic reticulum stress are also involved. These processes are involved in the development of liver fibrosis, cirrhosis, and HCC (Figure 1) [110].

Riazi et al. estimated that the overall prevalence of MASLD worldwide was 32.4% (95% CI: 29.9–34.9) in 2021 [111]. Its worldwide prevalence over time has since increased significantly and is now higher than previously estimated. It was reported that its prevalence in or before 2005 and in 2016 or later were 25.5% (20.1–31.0) and 37.8% (32.4–43.3), respectively (*p* = 0.013). Non-invasive tests identifying high-risk MASLD in primary care have been suggested, but the cost-effectiveness of such strategies remains uncertain [112]. The presence of MASLD or MASH is associated with high healthcare costs, and particularly in patients with MASH and T2DM [113].

HCC is still often diagnosed at an advanced stage, as indicated by its low rate of curative treatment, which then leads to a very poor prognosis, although therapies against advanced HCC have progressed compared to earlier ones [114,115]. HCC, which is derived from etiologies other than viral hepatitis, is associated with poor survival [116]. The detection of HCC at an early stage would also be needed for a better survival of patients with MASLD or MASH. New drugs for MASLD and MASH are being developed and some of them have been approved [117,118,119]. Genetic variants, including the PRS, may be one of the useful biomarkers for the early diagnosis of severe liver disease, and they may become candidates for therapies in patients with MASLD or MASH [98,104,120,121].

## 5. Conclusions

It may be important to perform multidisciplinary collaboration and cooperation involving hepatologists, geneticists, pharmacologists, and pathologists, thereby resolving complicated problems in MASLD and MASH patients. This narrative review highlights the future importance of the genetic susceptibility and PRS as predictive markers and personalized medicine for patients with MASLD or MASH. National genetic databases for MASLD and MASH should be established, and it will be necessary to incorporate the PRS into future guidelines.

## Figures and Tables

**Table 1:** Global frequency of PNPLA3 I148M and prevalence of MASLD.

Regions	PNPLA3 I148M (%)	MASLD (%)
Sub-Saharan Africa	12	13.5
Europe	23	25.1–44.4
South Asia	24–30	33.8
East Asia	35–45	28–33.1
Central and South America	~50%	~38.4
North America	~25	31.2

References [36,37,38]. PNPLA3, patatin-like phospholipase domain-containing protein; MASLD, metabolic dysfunction-associated steatotic liver disease.

**Table 2:** Positive or negative effects of the representative genetic variants on the progression of liver diseases in patients with MASLD and MASH.

*Genes*/Liver Diseases	Fat Accumulation/ALT Elevation	MASLD	MASH/Liver Fibrosis	Cirrhosis	HCC
*PNPLA3*	Positive	Positive	Positive	Positive	Positive
*HSD17B13*	Negative	Negative			
*GCKR*	Positive				
*TM6SF2*		Positive	Positive		Positive
*MBOAT7*		Positive	Positive		
*MERTK*			Negative	Negative	Negative

PNPLA3, patatin-like phospholipase domain-containing protein; HSD17B13, hydroxysteroid 17-beta dehydrogenase 13; GCKR, glucokinase regulator; TM6SF2, transmembrane 6 superfamily member 2; MBOAT7, membrane-bound O-acyltransferase domain-containing 7; MERTK, myeloid–epithelial–reproductive tyrosine kinase; MASLD, metabolic dysfunction-associated steatotic liver disease; MASH, metabolic dysfunction-associated steatohepatitis; HCC, hepatocellular carcinoma; negative, negative impact of genetic variants; positive, positive impact of genetic variants.

**Table 3:** Polygenic risk score (PRS) related to metabolic dysfunction-associated steatotic liver disease (MASLD) and metabolic dysfunction-associated steatohepatitis (MASH).

Authors, Years, Refs.	Number of Patients	Factors of PRS	Target of PRS	*p*-Values, etc.
***ALT Level***				
Larrieta-Carrasco E, et al., 2018 [84]	Baseline assessment from 2004 to 2006 (wave 1), Mexican Health Worker Cohort Study (MHWCS), n = ~4000; follow-up phase, n = 1855 (74%)	*ADIPOQ* rs17366743, *COL13A1* (rs7101190 and rs1227756), *PNPLA3* (rs3810622 and rs738409), *SAMM50* rs2143571	Higher ALT or AST levels	Mean ALT and AST levels significantly increased as a function of the number of risk alleles (quartiles) (*p* = 1.0 × 10^−9^ and 7.7 × 10^−11^, respectively), adjusted for age, sex, BMI, and admixture. PRS: elevated ALT levels, OR: 1.70 (95%CI: 1.41–2.05; *p* < 0.0001)
Stender S, et al., 2023 [85]	4018 children	Genome-wide ALT-PRS (*PNPLA3* rs738409, *TM6SF2* rs58542926, and *HSD17B13* rs72613567)	Plasma ALT levels	*p* for interaction between time and PRS on inverse normalized ALT = 1.5 × 10^−4^
***Fat Accumulation and Liver Fibrosis***				
Dongiovanni P, et al., 2018 [86]	Liver biopsy cohort (LBC), n = 1515; Swedish Obese Subjects Study (SOS), n = 3329; Dallas Heart Study (DHS), n = 4570	*PNPLA3* rs738409, *TM6SF2* rs58542926, *GCKR* rs1260326, *MBOAT7* rs641738	Hepatic fat (TG) accumulation	LBC: <10^−16^; (no PNPLA3), 1.2 × 10^−8^; LBC obese: 4.5 × 10^−6^; (no PNPLA3), 0.0059; (no TM6SF2), 0.0001; DHS: <2.2 × 10^−16^; (no PNPLA3), 5.8 × 10^−8^
Dongiovanni P, et al., 2018 [86]	Liver biopsy cohort (LBC), n = 1515	*PNPLA3* rs738409, *TM6SF2* rs58542926, *GCKR* rs1260326, *MBOAT7* rs641738	Liver fibrosis	*p* = 1.3 × 10^−14^
Åberg F, et al., 2023 [87]	5795 adults attending the Finnish Health 2000 Survey	PRS-5 based on variations in *PNPLA3*, *TM6SF2*, *GCKR*, *MBOAT7*, and *HSD17B13*	Liver fibrosis	Model 2: HR, 4.11; 95%CI, 1.24–13.64; *p* = 0.021; Model 3: HR, 5.05; 95%CI, 1.55–16.50; *p* = 0.007
***MASLD/NAFLD***				
Miao Z, et al., 2021 [88]	UK Biobank (UKB), training set (n = 99,823), test set (n = 34,833), and validation set (n = 5059).	PRS models (90 NAFLD GWAS loci)	NAFLD cases had a significantly higher PRS compared to control subjects	T= −7.89, *p* = 3.69 × 10^−15^, OR: 2.1
Ge X, et al., 2023 [89]	NAFLD in the UKB (N = 338,087)	PRS (variants in *PNPLA3*, *TM6SF2*, *MBOAT7*, and *GCKR*)	NAFLD	HR, 1.78 (95% CI, 1.60–1.98)
Fu T, et al., 2024 [90]	374,125 participants free of gastrointestinal disorders at baseline, including 19,719 (5.27%) with type 2 diabetes mellitus	PRS, using genetic variants ascertained to be strongly related to type 2 diabetes mellitus (*p* < 5 × 10^−8^)	NAFLD	Intermediate genetic risk: OR, 1.13 (95% CI, 1.02–1.25); high genetic risk: OR, 1.42 (95% CI, 1.26–1.59)
Mannisto VT, et al., 2021 [91]	Finnish population-based FINRISK and Health 2000 studies, n = 10,993 with NAFLD	PRS-5 (*PNPLA3*, *TM6SF2*, *MBOAT7*, *GCKR*, and *HSD17B13 genotype*)	The 20-year cumulative incidence of liver-related outcomes in NAFLD	4.3% in the high-PRS-5 group and 1.5% in the low-PRS-5 group (*p* < 0.001)
Giardoglou P, et al., 2024 [92]	4617 NAFLD/MRI-PDFF values from the UKB	PRS with 75 SNPs	Prediction of MASLD	Incremental R2 = 0.025, *p* = 0.00145
Kim S, et al., 2025 [93]	48,263 South Koreans (17,064 men and 31,199 women)	PRS related to dyslipidemia, using the “auto” mode of the PRS-continuous shrinkage method	Fatty liver index (FLI) and fibrotic NASH index (FNI) in men	AUROC [95% confidence intervals]: 0.704 [0.698–0.711]
***MASH/NASH***				
Gao F, et al., 2021 [94]	1070 Asian individuals with biopsy-confirmed NAFLD from 2 countries (China and South Korea)	Nomogram, including sex, metabolic syndrome, insulin resistance, AST ≥ 40 U/L, and *PNPLA3* (rs738409) and *HSD17B13* (rs72613567) genetic variants	NASH in the Eastern Asia region	High AUROCs (internal validation set: 0.80, 95% CI: 0.72–0.88; external validation cohort: 0.76, 95% CI: 0.72–0.80)
Bridi L, et al., 2024 [95]	382 patients with type 2 diabetes mellitus	PRS (the sum of risk alleles in *PNPLA3*, *TM6SF2*, and *SERPINA1* minus the protective variant in *HSD17B13*)	Prevalence of advanced fibrosis and cirrhosis	Higher PRSs were associated with an increased risk of cirrhosis (*p* = 0.037) and an increased risk of advanced cirrhosis among those with a fibrosis-4 index < 1.3 (*p* = 0.036)
***Severe liver disease, cirrhosis and HCC***				
De Vincentis, et al., 2022 [96]	UKB, n = 266,687	HFC-PRS (including *PNPLA3*, *TM6SF2*, *MBOAT7*, and *GCKR*); HFC-PRS (PRS2) (including only *PNPLA3* and *TM6SF2 variants*)	Severe liver disease (SLD), defined as a composite diagnosis of cirrhosis, decompensated liver disease, HCC, and/or liver transplantation	SLD: the HFC-PRS was highly associated with the risk of SLD in the overall population (age–sex-adjusted HR (aHR) for a 1 SD increase: 1.25; 95% CI, 1.16–1.35; *p* = 8.9 × 10^−9^)
Gellert-Kristensen H, et al., 2020 [97]	Copenhagen studies, n = 110,219; UKB, n = 334,276	*PNPLA3*, *TM6SF2*, and *HSD17B13 variants* (weighted by ALT effects)	Cirrhosis and HCC in Europeans	GRS 5-6: cirrhosis, OR: 12 (95% CI: 7.7–19); HCC, OR: 29 (95%CI: 17–51)
Bianco C, et al., 2021 [98]	NAFLD cohort, n = 2566; general population (UKB), n = 364,048	*PNPLA3-TM6SF2-GCKR-MBOAT7* and hepatic fat PRS (PRS-4), adjusted for *HSD17B13* (PRS-5)	HCC	Fatty liver: HFC-PRS, 4.4 × 10^−26^; PRS-5, 6.0 × 10^−27^; Fibrosis F3-F4: HFC-PRS, 9.5 × 10^−28^; PRS-5, 1.1 × 10^−30^; MASLD-HCC, HFC-PRS, 2.7 × 10^−14^; PRS-5, 1.6 × 10^−14^
Thomas CE, et al., 2022 [99]	24,333 participants of the Singapore Chinese Health Study (SCHS)	PRS for hepatic fat (HFC-PRS, including four SNPs: rs1260326 (*GCKR*), rs58542926 (*TM6SF2*), rs641738 (*MBOAT7*), and rs738409 (*PNPLA3*)); NAFLD-related PRS (NAFLD-PRS, including 12 SNPs)	Increased risk of HCC	HFC-PRS and NAFLD-PRS: HR of 2.39 (95% CI: 1.51, 3.78) and 1.77 (95% CI: 1.15, 2.73), respectively.
Thrift AP, et al., 2023 [100]	1644 patients with cirrhosis enrolled in two prospective cohort studies in the U.S.	PRS (high-risk variants in *PNPLA3-MBOAT7-TM6SF2-GCKR*)	HCC	Compared to cirrhosis patients in the lowest tertile of the PRS, those in the highest tertile had 2-fold higher risk of HCC (HR = 2.05; 95% CI, 1.22–3.44)
Xiao L, et al., 2024 [101]	435,306 from the UKB	PRS (16 genes)	Severe liver disease (SLD) and type 2 diabetes mellitus	HR, 3.15 (95% confidence interval, 2.54–3.90) for SLD; HR, 2.81 (2.60–3.03) for type 2 diabetes mellitus
***Others***				
Seko Y, et al., 2025 [46]	1304 Japanese patients with biopsy-proven MASLD	High-fat-content PRS (HFC-PRS), including *PNPLA3*, *TM6SF2*, *GCKR*, and *MBOAT7* genotypes	Prediction of liver-related events (LREs)	HRs for LRE: 10.72 in the high-risk group and 4.80 in the intermediate-risk group; HRs for prognosis: 8.74 in the high-risk group and 5.62 in the intermediate-risk group
Pirola CJ, et al., 2022 [102]	Microbial 16S rRNA reads from the livers of 116 individuals, categorized as non-NAFLD patients (n = 19) and patients with NAFLD (n = 44) and NASH (n = 53)	PRS (*PNPLA3*-rs738409, *TM6SF2*-rs58542926, *MBOAT7*-rs641738, *HSD17B13*-rs72613567, and *FGF21*-rs838133)	Abundance of the Tyzzerella genus—a member of the Firmicutes phylum and the Clostridia class—showed the strongest association with high PRS values (>4 risk alleles)	2.64-fold change differential abundance (*p* = 0.0019, FDR < 0.05)
Pirola CJ, et al., 2022 [102]	Microbial 16S rRNA reads from the liver of 116 individuals, categorized as non-NAFLD patients (n = 19) and patients with NAFL (n = 44) and NASH (n = 53)	PRS (*PNPLA3*-rs738409, *TM6SF2*-rs58542926, *MBOAT7*-rs641738, *HSD17B13*-rs72613567, and *FGF21*-rs838133)	Lactobacillus genus—a member of the Firmicutes phylum and Bacilli class—exhibited the strongest association with low PRS values (≤4 risk alleles)	0.89-fold change, *p* = 0.033, FDR < 0.05
Kafyra M, et al., 2023 [103]	2083 participants (the case-control Greek NAFLD study, the cross-sectional OSTEOS study, and the case-control THISEAS study)	PRS (16 SNPs)	BMI in Greek adults	R2 = 0.3241 (beta = 1.011, *p* = 4 × 10^−193^)
Guo HH, et al., 2025 [104]	UKB (N = 492,059)	PRS for UKB participants, using the PRS-CS method	Brain function scores	PRS for NAFLD, associated with declines in hand grip strength (β = −0.094, FDR-Q = 6.18 × 10^−5^) and usual walking speed (β = −0.136, FDR-Q = 2.80 × 10^−5^)

***Bold Oblique fonts*** mean the targets of PRSs. SNPs, single-nucleotide polymorphisms; ADIPOQ, adiponectin; COL13A1, collagen type XIII alpha 1 chain; PNPLA3, patatin-like phospholipase domain-containing protein 3; SAMM50, SAMM50 sorting and assembly machinery component; TM6SF2, transmembrane 6 superfamily member 2; HSD17B13, hydroxysteroid 17-beta dehydrogenase 13; GCKR, glucokinase regulator; MBOAT7, membrane-bound O-acyltransferase domain-containing 7; SERPINA1, serpin family A member 1; FGF21, fibroblast growth factor 21; ALT, alanine aminotransferase; AST, aspartate aminotransferase; BMI, body mass index; NAFLD, nonalcoholic fatty liver disease; NASH, nonalcoholic steatohepatitis; SLD, severe liver disease; HCC, hepatocellular carcinoma; OR, odds ratio; HR, hazard ratio; CI, confidence interval; AUROCs, area under the receiver operating characteristic curves.

## Data Availability

Not applicable.

## Abbreviations

The following abbreviations are used in this manuscript.

MASLD	metabolic dysfunction-associated steatotic liver disease
MASH	metabolic dysfunction-associated steatohepatitis
NAFLD	nonalcoholic fatty liver disease
NASH	nonalcoholic steatohepatitis
HCC	hepatocellular carcinoma
CVD	cardiovascular disease
GWAS	genome-wide association study
PRS	polygenic risk score
PNPLA3	patatin-like phospholipase domain-containing protein 3
TM6SF2	transmembrane 6 superfamily member 2
GCKR	glucokinase regulator
MBOAT7	membrane-bound O-acyltransferase domain-containing 7
MERTK	myeloid–epithelial–reproductive tyrosine kinase
HSD17B13	hydroxysteroid 17-beta dehydrogenase 13
ATGL	adipose triglyceride lipase
HSC	hepatic stellate cell
ALT	alanine aminotransferase
AST	aspartate aminotransferase
SNP	single-nucleotide polymorphism
CPN1	carboxypeptidase N subunit 1
ERLIN1	endoplasmic reticulum lipid raft-associated 1
CHUK/IKK-α	component of inhibitor of nuclear factor kappa B kinase (*NF-κß*) complex
SAMM50	SAMM50 sorting and assembly machinery component
IL	interleukin
DAPM	damage-associated molecular pattern
ASO	antisense oligonucleotide
siRNA	small interfering RNA
CRP	C-reactive protein
VLDL	very-low-density lipoprotein
HTGC	hepatic triglyceride content
DHS	Dallas Heart Study
LDL	low-density lipoprotein
PMBB	Penn Medicine Biobank
WES	whole-exome sequence
UKB	UK Biobank
OR	odds ratio
HFHC	high-fat/high-cholesterol
GK	glucokinase
GKRP	GK regulatory protein
TLR	toll-like receptor
ADAM17	ADAM metallopeptidase domain 17 (ADAM17)
ENPP1	ectonucleotide pyrophosphatase/phosphodiesterase
MTTP/MTP	microsomal triglyceride transfer protein
UCP2	uncoupling protein 2
ROS	reactive oxygen species
COL13A1	collagen type XIII alpha 1 chain
HOMA-IR	homeostatic model assessment for insulin resistance
HFC-PRS	PRS for hepatic fat

## References

[1] Younossi Z.M., Golabi P., Price J.K., Owrangi S., Gundu-Rao N., Satchi R., Paik J.M. (2024). The Global Epidemiology of Nonalcoholic Fatty Liver Disease and Nonalcoholic Steatohepatitis Among Patients With Type 2 Diabetes. Clin. Gastroenterol. Hepatol..

[2] Owrangi S., Paik J.M., Golabi P., de Avila L., Hashida R., Nader A., Paik A., Henry L., Younossi Z.M. (2025). Meta-Analysis: Global Prevalence and Mortality of Cirrhosis in Metabolic Dysfunction-Associated Steatotic Liver Disease. Aliment. Pharmacol. Ther..

[3] Eslam M., Sanyal A.J., George J., International Consensus Panel (2020). MAFLD: A Consensus-Driven Proposed Nomenclature for Metabolic Associated Fatty Liver Disease. Gastroenterology.

[4] Eslam M., Newsome P.N., Sarin S.K., Anstee Q.M., Targher G., Romero-Gomez M., Zelber-Sagi S., Wai-Sun Wong V., Dufour J.F., Schattenberg J.M. (2020). A new definition for metabolic dysfunction-associated fatty liver disease: An international expert consensus statement. J. Hepatol..

[5] Kanwal F., Neuschwander-Tetri B.A., Loomba R., Rinella M.E. (2024). Metabolic dysfunction-associated steatotic liver disease: Update and impact of new nomenclature on the American Association for the Study of Liver Diseases practice guidance on nonalcoholic fatty liver disease. Hepatology.

[6] Ramírez-Mejía M.M., Jiménez-Gutiérrez C., Eslam M., George J., Méndez-Sánchez N. (2024). Breaking new ground: MASLD vs. MAFLD-which holds the key for risk stratification?. Hepatol. Int..

[7] Fan J.G., Xu X.Y., Yang R.X., Nan Y.M., Wei L., Jia J.D., Zhuang H., Shi J.P., Li X.Y., Sun C. (2024). Guideline for the Prevention and Treatment of Metabolic Dysfunction-associated Fatty Liver Disease (Version 2024). J. Clin. Transl. Hepatol..

[8] Kanda T., Goto T., Hirotsu Y., Masuzaki R., Moriyama M., Omata M. (2020). Molecular Mechanisms: Connections between Nonalcoholic Fatty Liver Disease, Steatohepatitis and Hepatocellular Carcinoma. Int. J. Mol. Sci..

[9] Eslam M., Fan J.G., Yu M.L., Wong V.W., Cua I.H., Liu C.J., Tanwandee T., Gani R., Seto W.K., Alam S. (2025). The Asian Pacific association for the study of the liver clinical practice guidelines for the diagnosis and management of metabolic dysfunction-associated fatty liver disease. Hepatol. Int..

[10] Ascha M.S., Hanouneh I.A., Lopez R., Tamimi T.A., Feldstein A.F., Zein N.N. (2010). The incidence and risk factors of hepatocellular carcinoma in patients with nonalcoholic steatohepatitis. Hepatology.

[11] Ertle J., Dechêne A., Sowa J.P., Penndorf V., Herzer K., Kaiser G., Schlaak J.F., Gerken G., Syn W.K., Canbay A. (2011). Non-alcoholic fatty liver disease progresses to hepatocellular carcinoma in the absence of apparent cirrhosis. Int. J. Cancer.

[12] Niederseer D., Wernly B., Aigner E., Stickel F., Datz C. (2021). NAFLD and Cardiovascular Diseases: Epidemiological, Mechanistic and Therapeutic Considerations. J. Clin. Med..

[13] Theodorakis N., Nikolaou M. (2025). From Cardiovascular-Kidney-Metabolic Syndrome to Cardiovascular-Renal-Hepatic-Metabolic Syndrome: Proposing an Expanded Framework. Biomolecules.

[14] Rinella M.E., Neuschwander-Tetri B.A., Siddiqui M.S., Abdelmalek M.F., Caldwell S., Barb D., Kleiner D.E., Loomba R. (2023). AASLD Practice Guidance on the clinical assessment and management of nonalcoholic fatty liver disease. Hepatology.

[15] Tanaka Y., Nishida N., Sugiyama M., Kurosaki M., Matsuura K., Sakamoto N., Nakagawa M., Korenaga M., Hino K., Hige S. (2009). Genome-wide association of IL28B with response to pegylated interferon-alpha and ribavirin therapy for chronic hepatitis C. Nat. Genet..

[16] Suppiah V., Moldovan M., Ahlenstiel G., Berg T., Weltman M., Abate M.L., Bassendine M., Spengler U., Dore G.J., Powell E. (2009). IL28B is associated with response to chronic hepatitis C interferon-alpha and ribavirin therapy. Nat. Genet..

[17] Thomas D.L., Thio C.L., Martin M.P., Qi Y., Ge D., O’Huigin C., Kidd J., Kidd K., Khakoo S.I., Alexander G. (2009). Genetic variation in IL28B and spontaneous clearance of hepatitis C virus. Nature.

[18] Torkamani A., Wineinger N.E., Topol E.J. (2018). The personal and clinical utility of polygenic risk scores. Nat. Rev. Genet..

[19] Lewis C.M., Vassos E. (2020). Polygenic risk scores: From research tools to clinical instruments. Genome Med..

[20] Pingitore P., Romeo S. (2019). The role of PNPLA3 in health and disease. Biochim. Biophys. Acta Mol. Cell Biol. Lipids.

[21] Huang Y., He S., Li J.Z., Seo Y.K., Osborne T.F., Cohen J.C., Hobbs H.H. (2010). A feed-forward loop amplifies nutritional regulation of PNPLA3. Proc. Natl. Acad. Sci. USA.

[22] Pirazzi C., Valenti L., Motta B.M., Pingitore P., Hedfalk K., Mancina R.M., Burza M.A., Indiveri C., Ferro Y., Montalcini T. (2014). PNPLA3 has retinyl-palmitate lipase activity in human hepatic stellate cells. Hum. Mol. Genet..

[23] Wilson P.A., Gardner S.D., Lambie N.M., Commans S.A., Crowther D.J. (2006). Characterization of the human patatin-like phospholipase family. J. Lipid Res..

[24] Dong X.C. (2019). PNPLA3-A Potential Therapeutic Target for Personalized Treatment of Chronic Liver Disease. Front. Med..

[25] Johnson S.M., Bao H., McMahon C.E., Chen Y., Burr S.D., Anderson A.M., Madeyski-Bengtson K., Lindén D., Han X., Liu J. (2024). PNPLA3 is a triglyceride lipase that mobilizes polyunsaturated fatty acids to facilitate hepatic secretion of large-sized very low-density lipoprotein. Nat. Commun..

[26] Takahashi Y., Dungubat E., Kusano H., Fukusato T. (2023). Pathology and Pathogenesis of Metabolic Dysfunction-Associated Steatotic Liver Disease-Associated Hepatic Tumors. Biomedicines.

[27] Yuan X., Waterworth D., Perry J.R., Lim N., Song K., Chambers J.C., Zhang W., Vollenweider P., Stirnadel H., Johnson T. (2008). Population-based genome-wide association studies reveal six loci influencing plasma levels of liver enzymes. Am. J. Hum. Genet..

[28] Bruschi F.V., Tardelli M., Claudel T., Trauner M. (2017). PNPLA3 expression and its impact on the liver: Current perspectives. Hepat. Med..

[29] Romeo S., Kozlitina J., Xing C., Pertsemlidis A., Cox D., Pennacchio L.A., Boerwinkle E., Cohen J.C., Hobbs H.H. (2008). Genetic variation in PNPLA3 confers susceptibility to nonalcoholic fatty liver disease. Nat. Genet..

[30] Schilcher K., Dayoub R., Kubitza M., Riepl J., Klein K., Buechler C., Melter M., Weiss T.S. (2023). Saturated Fat-Mediated Upregulation of IL-32 and CCL20 in Hepatocytes Contributes to Higher Expression of These Fibrosis-Driving Molecules in MASLD. Int. J. Mol. Sci..

[31] BasuRay S., Wang Y., Smagris E., Cohen J.C., Hobbs H.H. (2019). Accumulation of PNPLA3 on lipid droplets is the basis of associated hepatic steatosis. Proc. Natl. Acad. Sci. USA.

[32] Wang Y., Kory N., BasuRay S., Cohen J.C., Hobbs H.H. (2019). PNPLA3, CGI-58, and Inhibition of Hepatic Triglyceride Hydrolysis in Mice. Hepatology.

[33] Bril F., Kalavalapalli S., Lomonaco R., Frye R., Godinez Leiva E., Cusi K. (2024). Insulin resistance is an integral feature of MASLD even in the presence of PNPLA3 variants. JHEP Rep..

[34] Maiorana F., Neschuk M., Caronia M.V., Elizondo K., Robledo M.L., Schneider A., Veron G., Zapata P.D., Barreyro F.J. (2024). The interplay between Helicobacter pylori infection and rs738409 PNPLA3 in metabolic dysfunction-associated steatotic liver disease. PLoS ONE.

[35] Castanho Martins M., Dixon E.D., Lupo G., Claudel T., Trauner M., Rombouts K. (2025). Role of PNPLA3 in Hepatic Stellate Cells and Hepatic Cellular Crosstalk. Liver Int..

[36] Lazo M., Xie J., Alvarez C.S., Parisi D., Yang S., Rivera-Andrade A., Kroker-Lobos M.F., Groopman J.D., Guallar E., Ramirez-Zea M. (2022). Frequency of the PNPLA3 rs738409 polymorphism and other genetic loci for liver disease in a Guatemalan adult population. Liver Int..

[37] Hassan M.M., Li D., Han Y., Byun J., Hatia R.I., Long E., Choi J., Kelley R.K., Cleary S.P., Lok A.S. (2024). Genome-wide association study identifies high-impact susceptibility loci for HCC in North America. Hepatology.

[38] Kozlitina J., Sookoian S. (2025). Global Epidemiological Impact of PNPLA3 I148M on Liver Disease. Liver Int..

[39] Caddeo A., Romeo S. (2025). Precision medicine and nucleotide-based therapeutics to treat steatotic liver disease. Clin. Mol. Hepatol..

[40] Armisen J., Rauschecker M., Sarv J., Liljeblad M., Wernevik L., Niazi M., Knöchel J., Eklund O., Sandell T., Sherwood J. (2025). AZD2693, a PNPLA3 antisense oligonucleotide, for the treatment of MASH in 148M homozygous participants: Two randomized phase I trials. J. Hepatol..

[41] Lindén D., Tesz G., Loomba R. (2025). Targeting PNPLA3 to Treat MASH and MASH Related Fibrosis and Cirrhosis. Liver Int..

[42] Xia M., Varmazyad M., Pla-Palacín I., Gavlock D.C., DeBiasio R., LaRocca G., Reese C., Florentino R.M., Faccioli L.A.P., Brown J.A. (2024). Comparison of wild-type and high-risk PNPLA3 variants in a human biomimetic liver microphysiology system for metabolic dysfunction-associated steatotic liver disease precision therapy. Front. Cell Dev. Biol..

[43] Kozlitina J., Smagris E., Stender S., Nordestgaard B.G., Zhou H.H., Tybjærg-Hansen A., Vogt T.F., Hobbs H.H., Cohen J.C. (2014). Exome-wide association study identifies a TM6SF2 variant that confers susceptibility to nonalcoholic fatty liver disease. Nat. Genet..

[44] Mahdessian H., Taxiarchis A., Popov S., Silveira A., Franco-Cereceda A., Hamsten A., Eriksson P., van’t Hooft F. (2014). TM6SF2 is a regulator of liver fat metabolism influencing triglyceride secretion and hepatic lipid droplet content. Proc. Natl. Acad. Sci. USA.

[45] Huang H.Y.R., Vitali C., Zhang D., Hand N.J., Phillips M.C., Creasy K.T., Scorletti E., Park J., Schneider K.M., Regeneron Centre (2024). Deep metabolic phenotyping of humans with protein-altering variants in TM6SF2 using a genome-first approach. JHEP Rep..

[46] Seko Y., Yamaguchi K., Shima T., Iwaki M., Takahashi H., Kawanaka M., Tanaka S., Mitsumoto Y., Yoneda M., Nakajima A. (2025). Clinical Utility of Genetic Variants in PNPLA3 and TM6SF2 to Predict Liver-Related Events in Metabolic Dysfunction-Associated Steatotic Liver Disease. Liver Int..

[47] Zhang Y., Xie M., Wen J., Liang C., Song Q., Liu W., Liu Y., Song Y., Lau H.C.H., Cheung A.H. (2025). Hepatic TM6SF2 activates antitumour immunity to suppress metabolic dysfunction-associated steatotic liver disease-related hepatocellular carcinoma and boosts immunotherapy. Gut.

[48] Wang K., Shi M., Luk A.O.Y., Kong A.P.S., Ma R.C.W., Li C., Chen L., Chow E., Chan J.C.N. (2024). Impaired GK-GKRP interaction rather than direct GK activation worsens lipid profiles and contributes to long-term complications: A Mendelian randomization study. Cardiovasc. Diabetol..

[49] Beer N.L., Tribble N.D., McCulloch L.J., Roos C., Johnson P.R., Orho-Melander M., Gloyn A.L. (2009). The P446L variant in GCKR associated with fasting plasma glucose and triglyceride levels exerts its effect through increased glucokinase activity in liver. Hum. Mol. Genet..

[50] Maffeis C., Piona C., Morandi A., Marigliano M., Morotti E., Mancioppi V., Caiazza E., Zusi C., Emiliani F., Mantovani A. (2024). Glycaemic control metrics and metabolic dysfunction-associated steatotic liver disease in children and adolescents with type 1 diabetes. Diabetes Obes. Metab..

[51] Santoro N., Caprio S., Pierpont B., Van Name M., Savoye M., Parks E.J. (2015). Hepatic De Novo Lipogenesis in Obese Youth Is Modulated by a Common Variant in the GCKR Gene. J. Clin. Endocrinol. Metab..

[52] Santoro N., Zhang C.K., Zhao H., Pakstis A.J., Kim G., Kursawe R., Dykas D.J., Bale A.E., Giannini C., Pierpont B. (2012). Variant in the glucokinase regulatory protein (GCKR) gene is associated with fatty liver in obese children and adolescents. Hepatology.

[53] Hernaez R., McLean J., Lazo M., Brancati F.L., Hirschhorn J.N., Borecki I.B., Harris T.B., Nguyen T., Kamel I.R., Genetics of Obesity-Related Liver Disease (GOLD) Consortium (2013). Association between variants in or near PNPLA3, GCKR, and PPP1R3B with ultrasound-defined steatosis based on data from the third National Health and Nutrition Examination Survey. Clin. Gastroenterol. Hepatol..

[54] Mancina R.M., Dongiovanni P., Petta S., Pingitore P., Meroni M., Rametta R., Borén J., Montalcini T., Pujia A., Wiklund O. (2016). The MBOAT7-TMC4 Variant rs641738 Increases Risk of Nonalcoholic Fatty Liver Disease in Individuals of European Descent. Gastroenterology.

[55] Buch S., Stickel F., Trépo E., Way M., Herrmann A., Nischalke H.D., Brosch M., Rosendahl J., Berg T., Ridinger M. (2015). A genome-wide association study confirms PNPLA3 and identifies TM6SF2 and MBOAT7 as risk loci for alcohol-related cirrhosis. Nat. Genet..

[56] Thabet K., Asimakopoulos A., Shojaei M., Romero-Gomez M., Mangia A., Irving W.L., Berg T., Dore G.J., Grønbæk H., Sheridan D. (2016). MBOAT7 rs641738 increases risk of liver inflammation and transition to fibrosis in chronic hepatitis C. Nat. Commun..

[57] Alharthi J., Bayoumi A., Thabet K., Pan Z., Gloss B.S., Latchoumanin O., Lundberg M., Twine N.A., McLeod D., Alenizi S. (2022). A metabolic associated fatty liver disease risk variant in MBOAT7 regulates toll like receptor induced outcomes. Nat. Commun..

[58] Moore M.P., Wang X., Kennelly J.P., Shi H., Ishino Y., Kano K., Aoki J., Cherubini A., Ronzoni L., Guo X. (2025). Low MBOAT7 expression, a genetic risk for MASH, promotes a profibrotic pathway involving hepatocyte TAZ upregulation. Hepatology.

[59] Yang Y., Chen X., Zhang H., Yang G., Zhu X., Si X., Chen F., Zhao Y., Jin F., Lu J. (2025). The correlation between the polymorphism of lysolecithin acyltransferase (MBOAT7) rs641738 and liver fibrosis. Pers. Med..

[60] Petta S., Valenti L., Marra F., Grimaudo S., Tripodo C., Bugianesi E., Cammà C., Cappon A., Di Marco V., Di Maira G. (2016). MERTK rs4374383 polymorphism affects the severity of fibrosis in non-alcoholic fatty liver disease. J. Hepatol..

[61] Cai B., Dongiovanni P., Corey K.E., Wang X., Shmarakov I.O., Zheng Z., Kasikara C., Davra V., Meroni M., Chung R.T. (2020). Macrophage MerTK Promotes Liver Fibrosis in Nonalcoholic Steatohepatitis. Cell Metab..

[62] Wang X., Cai B. (2020). MerTK, a risk factor for NASH fibrosis. Aging.

[63] Tutusaus A., Morales A., García de Frutos P., Marí M. (2024). GAS6/TAM Axis as Therapeutic Target in Liver Diseases. Semin. Liver Dis..

[64] Grøndal S.M., Tutusaus A., Boix L., Reig M., Blø M., Hodneland L., Gausdal G., Jackson A., Garcia de Frutos P., Lorens J.B. (2024). Dynamic changes in immune cell populations by AXL kinase targeting diminish liver inflammation and fibrosis in experimental MASH. Front. Immunol..

[65] Abul-Husn N.S., Cheng X., Li A.H., Xin Y., Schurmann C., Stevis P., Liu Y., Kozlitina J., Stender S., Wood G.C. (2018). A Protein-Truncating HSD17B13 Variant and Protection from Chronic Liver Disease. N. Engl. J. Med..

[66] Demirtas C.O., Yilmaz Y. (2024). Decoding 17-Beta-hydroxysteroid Dehydrogenase 13: A Multifaceted Perspective on Its Role in Hepatic Steatosis and Associated Disorders. J. Clin. Transl. Hepatol..

[67] Mahmood S., Morrice N., Thompson D., Milanizadeh S., Wilson S., Whitfield P.D., Mcilroy G.D., Rochford J.J., Mody N. (2025). Hydroxysteroid 17beta-dehydrogenase 13 (Hsd17b13) knockdown attenuates liver steatosis in high-fat diet obese mice. Exp. Physiol..

[68] Hudert C.A., Selinski S., Rudolph B., Bläker H., Loddenkemper C., Thielhorn R., Berndt N., Golka K., Cadenas C., Reinders J. (2019). Genetic determinants of steatosis and fibrosis progression in paediatric non-alcoholic fatty liver disease. Liver Int..

[69] Liu X., Chen S., Liu X., Wu X., Jiang X., Li Y., Yang Z. (2025). Enpp1 ameliorates MAFLD by regulating hepatocyte lipid metabolism through the AMPK/PPARα signaling pathway. Cell Biosci..

[70] Uygun A., Ozturk K., Demirci H., Oztuna A., Eren F., Kozan S., Yilmaz Y., Kurt O., Turker T., Vatansever S. (2017). The association of nonalcoholic fatty liver disease with genetic polymorphisms: A multicenter study. Eur. J. Gastroenterol. Hepatol..

[71] Yuan C., Lu L., An B., Jin W., Dong Q., Xin Y., Xuan S. (2015). Association Between LYPLAL1 rs12137855 Polymorphism With Ultrasound-Defined Non-Alcoholic Fatty Liver Disease in a Chinese Han Population. Hepat. Mon..

[72] Saliba-Gustafsson P., Justesen J.M., Ranta A., Sharma D., Bielczyk-Maczynska E., Li J., Najmi L.A., Apodaka M., Aspichueta P., Björck H.M. (2024). A functional genomic framework to elucidate novel causal metabolic dysfunction-associated fatty liver disease genes. Hepatology.

[73] Filip R., Bélanger É., Chen X., Lefebvre D., Uguccioni S.M., Pezacki J.P. (2025). LYPLAL1 enzyme activity is linked to hepatic glucose metabolism. Biochem. Biophys. Res. Commun..

[74] Chouik Y., Di Filippo M., Radenne S., Dumortier J., Moulin P., Levrero M. (2024). Combination of heterozygous APOB gene mutation with PNPLA3 and TM6SF2 variants promotes steatotic liver disease, cirrhosis and HCC development. Liver Int..

[75] Schneider C.V., Hehl L., Creasy K.T., Vitali C., Vell M.S., Vujkovic M., Park J., Scorletti E., Seeling K.S., Rendel M.D. (2023). A coding variant in the microsomal triglyceride transfer protein reduces both hepatic steatosis and plasma lipids. Aliment. Pharmacol. Ther..

[76] Haas M.E., Pirruccello J.P., Friedman S.N., Wang M., Emdin C.A., Ajmera V.H., Simon T.G., Homburger J.R., Guo X., Budoff M. (2021). Machine learning enables new insights into genetic contributions to liver fat accumulation. Cell Genom..

[77] Valenti L., Motta B.M., Alisi A., Sartorelli R., Buonaiuto G., Dongiovanni P., Rametta R., Pelusi S., Fargion S., Nobili V. (2012). LPIN1 rs13412852 polymorphism in pediatric nonalcoholic fatty liver disease. J. Pediatr. Gastroenterol. Nutr..

[78] Liukkonen V., Semenova M., Hyvärinen K., Lauronen J., Partanen J., Arola J., Nordin A., Färkkilä M., Åberg F. (2025). Genetic Risk Factors for Steatotic Liver Disease After Liver Transplantation. Liver Int..

[79] Cortez-Pinto H., Zhi Lin H., Qi Yang S., Odwin Da Costa S., Diehl A.M. (1999). Lipids up-regulate uncoupling protein 2 expression in rat hepatocytes. Gastroenterology.

[80] Maamari D.J., Abou-Karam R., Fahed A.C. (2025). Polygenic Risk Scores in Human Disease. Clin. Chem..

[81] Choi S.W., Mak T.S., O’Reilly P.F. (2020). Tutorial: A guide to performing polygenic risk score analyses. Nat. Protoc..

[82] Loh M., Chambers J.C. (2023). Polygenic risk scores for complex diseases: Where are we now?. Singap. Med. J..

[83] Lennon N.J., Kottyan L.C., Kachulis C., Abul-Husn N.S., Arias J., Belbin G., Below J.E., Berndt S.I., Chung W.K., Cimino J.J. (2024). Selection, optimization and validation of ten chronic disease polygenic risk scores for clinical implementation in diverse US populations. Nat. Med..

[84] Larrieta-Carrasco E., Flores Y.N., Macías-Kauffer L.R., Ramírez-Palacios P., Quiterio M., Ramírez-Salazar E.G., León-Mimila P., Rivera-Paredez B., Cabrera-Álvarez G., Canizales-Quinteros S. (2018). Genetic variants in COL13A1, ADIPOQ and SAMM50, in addition to the PNPLA3 gene, confer susceptibility to elevated transaminase levels in an admixed Mexican population. Exp. Mol. Pathol..

[85] Stender S., Davey Smith G., Richardson T.G. (2023). Genetic variation and elevated liver enzymes during childhood, adolescence and early adulthood. Int. J. Epidemiol..

[86] Dongiovanni P., Stender S., Pietrelli A., Mancina R.M., Cespiati A., Petta S., Pelusi S., Pingitore P., Badiali S., Maggioni M. (2018). Causal relationship of hepatic fat with liver damage and insulin resistance in nonalcoholic fatty liver. J. Intern. Med..

[87] Åberg F., Saarinen K., Jula A., Lundqvist A., Vihervaara T., Erlund I., Färkkilä M. (2023). Combined use of the ELF test and CLivD score improves prediction of liver-related outcomes in the general population. Liver Int..

[88] Miao Z., Garske K.M., Pan D.Z., Koka A., Kaminska D., Männistö V., Sinsheimer J.S., Pihlajamäki J., Pajukanta P. (2021). Identification of 90 NAFLD GWAS loci and establishment of NAFLD PRS and causal role of NAFLD in coronary artery disease. HGG Adv..

[89] Ge X., Wang X., Yan Y., Zhang L., Yu C., Lu J., Xu X., Gao J., Liu M., Jiang T. (2023). Behavioural activity pattern, genetic factors, and the risk of nonalcoholic fatty liver disease: A prospective study in the UK Biobank. Liver Int..

[90] Fu T., Sun Y., Lu S., Zhao J., Dan L., Shi W., Chen J., Chen Y., Li X. (2024). Risk Assessment for Gastrointestinal Diseases via Clinical Dimension and Genome-Wide Polygenic Risk Scores of Type 2 Diabetes: A Population-Based Cohort Study. Diabetes Care.

[91] Männistö V.T., Salomaa V., Färkkilä M., Jula A., Männistö S., Erlund I., Sundvall J., Lundqvist A., Perola M., Åberg F. (2021). Incidence of liver-related morbidity and mortality in a population cohort of non-alcoholic fatty liver disease. Liver Int..

[92] Giardoglou P., Gavra I., Amanatidou A.I., Kalafati I.P., Symianakis P., Kafyra M., Moulos P., Dedoussis G.V. (2024). Development of a Polygenic Risk Score for Metabolic Dysfunction-Associated Steatotic Liver Disease Prediction in UK Biobank. Genes.

[93] Kim S., Yoo H.Y. (2025). Sex differences in predicting dyslipidemia using polygenic risk score with fatty liver index and fibrotic nonalcoholic steatohepatitis index. Sci. Rep..

[94] Gao F., Zheng K.I., Chen S.D., Lee D.H., Wu X.X., Wang X.D., Targher G., Byrne C.D., Chen Y.P., Kim W. (2021). Individualized Polygenic Risk Score Identifies NASH in the Eastern Asia Region: A Derivation and Validation Study. Clin. Transl. Gastroenterol..

[95] Bridi L., Agrawal S., Tesfai K., Madamba E., Bettencourt R., Richards L.M., Khera A.V., Loomba R., Ajmera V. (2024). The impact of genetic risk on the prevalence of advanced fibrosis and cirrhosis in prospectively assessed patients with type 2 diabetes. Aliment. Pharmacol. Ther..

[96] De Vincentis A., Tavaglione F., Jamialahmadi O., Picardi A., Antonelli Incalzi R., Valenti L., Romeo S., Vespasiani-Gentilucci U. (2022). A Polygenic Risk Score to Refine Risk Stratification and Prediction for Severe Liver Disease by Clinical Fibrosis Scores. Clin. Gastroenterol. Hepatol..

[97] Gellert-Kristensen H., Richardson T.G., Davey Smith G., Nordestgaard B.G., Tybjaerg-Hansen A., Stender S. (2020). Combined Effect of PNPLA3, TM6SF2, and HSD17B13 Variants on Risk of Cirrhosis and Hepatocellular Carcinoma in the General Population. Hepatology.

[98] Bianco C., Jamialahmadi O., Pelusi S., Baselli G., Dongiovanni P., Zanoni I., Santoro L., Maier S., Liguori A., Meroni M. (2021). Non-invasive stratification of hepatocellular carcinoma risk in non-alcoholic fatty liver using polygenic risk scores. J. Hepatol..

[99] Thomas C.E., Diergaarde B., Kuipers A.L., Adibi J.J., Luu H.N., Chang X., Dorajoo R., Heng C.K., Khor C.C., Wang R. (2022). NAFLD polygenic risk score and risk of hepatocellular carcinoma in an East Asian population. Hepatol. Commun..

[100] Thrift A.P., Kanwal F., Liu Y., Khaderi S., Singal A.G., Marrero J.A., Loo N., Asrani S.K., Luster M., Al-Sarraj A. (2023). Risk stratification for hepatocellular cancer among patients with cirrhosis using a hepatic fat polygenic risk score. PLoS ONE.

[101] Xiao L., Li Y., Hong C., Ma P., Zhu H., Cui H., Zou X., Wang J., Li R., He J. (2024). Polygenic risk score of metabolic dysfunction-associated steatotic liver disease amplifies the health impact on severe liver disease and metabolism-related outcomes. J. Transl. Med..

[102] Pirola C.J., Salatino A., Quintanilla M.F., Castaño G.O., Garaycoechea M., Sookoian S. (2022). The influence of host genetics on liver microbiome composition in patients with NAFLD. EBioMedicine.

[103] Kafyra M., Kalafati I.P., Dimitriou M., Grigoriou E., Kokkinos A., Rallidis L., Kolovou G., Trovas G., Marouli E., Deloukas P. (2023). Robust Bioinformatics Approaches Result in the First Polygenic Risk Score for BMI in Greek Adults. J. Pers. Med..

[104] Guo H.H., Gao P.Y., Zhang W., Fu Y., Chi H.C., Zhang Z.H., Han S.L., Han B.L., Zhang Y.Y., Xu W. (2025). Liver Diseases and Brain Disorders: Genetic Mechanisms and Biomarker Pathways in a Prospective Cohort Study From the UK Biobank. J. Neurochem..

[105] Faccioli L.A.P., Cetin Z., Kocas-Kilicarslan Z.N., Ortiz K., Sun Y., Hu Z., Kurihara T., Tafaleng E.N., Florentino R.M., Wang Z. (2023). Evaluation of Human Hepatocyte Drug Metabolism Carrying High-Risk or Protection-Associated Liver Disease Genetic Variants. Int. J. Mol. Sci..

[106] European Association for the Study of the Liver (EASL), European Association for the Study of Diabetes (EASD), European Association for the Study of Obesity (EASO) (2024). EASL-EASD-EASO Clinical Practice Guidelines on the management of metabolic dysfunction-associated steatotic liver disease (MASLD). J. Hepatol..

[107] Moonlisarn K., Somnark P., Boonkaew B., Bunchorntavakul C., Tangkijvanich P. (2024). Interaction Between PNPLA3 and SIRT5 Genetic Variants in Association with Liver Fibrosis Severity in Patients with Metabolic Dysfunction-Associated Steatotic Liver Disease. Genes.

[108] Fabbrini E., Rady B., Koshkina A., Jeon J.Y., Ayyar V.S., Gargano C., DiProspero N., Wendel S., Hegge J., Hamilton H. (2024). Phase 1 Trials of PNPLA3 siRNA in I148M Homozygous Patients with MAFLD. N. Engl. J. Med..

[109] Sato S., Iino C., Sasada T., Furusawa K., Yoshida K., Sawada K., Mikami T., Fukuda S., Nakaji S., Sakuraba H. (2025). A 4-year cohort study of the effects of PNPLA3 rs738409 genotypes on liver fat and fibrosis and gut microbiota in a non-fatty liver population. Environ. Health Prev. Med..

[110] Kanda T., Matsuoka S., Yamazaki M., Shibata T., Nirei K., Takahashi H., Kaneko T., Fujisawa M., Higuchi T., Nakamura H. (2018). Apoptosis and non-alcoholic fatty liver diseases. World J. Gastroenterol..

[111] Riazi K., Azhari H., Charette J.H., Underwood F.E., King J.A., Afshar E.E., Swain M.G., Congly S.E., Kaplan G.G., Shaheen A.A. (2022). The prevalence and incidence of NAFLD worldwide: A systematic review and meta-analysis. Lancet Gastroenterol. Hepatol..

[112] Younossi Z.M., Paik J.M., Henry L., Pollock R.F., Stepanova M., Nader F. (2025). Economic evaluation of non-invasive test pathways for high-risk metabolic dysfunction-associated steatotic liver disease (MASLD) in the United Kingdom (UK). Ann. Hepatol..

[113] Díaz Carnicero J., Saurí-Ferrer I., Redon J., Navarro J., Fernández G., Hurtado C., Ferreira K., Alvarez-Ortega C., Gómez A., Martos-Rodríguez C.J. (2025). Clinical and Economic Burden of Metabolic Dysfunction-Associated Steatotic Liver Disease (MASLD) in a Spanish Mediterranean Region: A Population-Based Study. J. Clin. Med..

[114] Finn R.S., Qin S., Ikeda M., Galle P.R., Ducreux M., Kim T.Y., Kudo M., Breder V., Merle P., Kaseb A.O. (2020). Atezolizumab plus bevacizumab in unresectable hepatocellular carcinoma. N. Engl. J. Med..

[115] Rimassa L., Chan S.L., Sangro B., Lau G., Kudo M., Reig M., Breder V., Ryu M.H., Ostapenko Y., Sukeepaisarnjaroen W. (2025). Five-year overall survival update from the HIMALAYA study of tremelimumab plus durvalumab in unresectable HCC. J. Hepatol..

[116] Mathurin P., de Zélicourt M., Laurendeau C., Dhaoui M., Kelkouli N., Blanc J.F. (2023). Treatment patterns, risk factors and outcomes for patients with newly diagnosed hepatocellular carcinoma in France: A retrospective database analysis. Clin. Res. Hepatol. Gastroenterol..

[117] Harrison S.A., Taub R., Neff G.W., Lucas K.J., Labriola D., Moussa S.E., Alkhouri N., Bashir M.R. (2023). Resmetirom for nonalcoholic fatty liver disease: A randomized, double-blind, placebo-controlled phase 3 trial. Nat. Med..

[118] Harrison S.A., Bedossa P., Guy C.D., Schattenberg J.M., Loomba R., Taub R., Labriola D., Moussa S.E., Neff G.W., Rinella M.E. (2024). A Phase 3, Randomized, Controlled Trial of Resmetirom in NASH with Liver Fibrosis. N. Engl. J. Med..

[119] Souza M., Al-Sharif L., Antunes V.L.J., Huang D.Q., Loomba R. (2025). Comparison of pharmacological therapies in metabolic dysfunction-associated steatohepatitis for fibrosis regression and MASH resolution: Systematic review and network meta-analysis. Hepatology.

[120] Seko Y., Yamaguchi K., Shima T., Tanaka S., Shirono T., Takahashi Y., Takeuchi K., Kataoka S., Moriguchi M., Okanoue T. (2024). Prognostic performance of a two-step method using the Fibro-Scope system for metabolic dysfunction-associated steatotic liver disease. Hepatol. Res..

[121] Seko Y., Lin H., Wong V.W., Okanoue T. (2025). Impact of PNPLA3 in Lean Individuals and in Cryptogenic Steatotic Liver Disease. Liver Int..

